# Revisiting the Dexamethasone Suppression Test in unipolar major depression: an exploratory study

**DOI:** 10.1186/1744-859X-7-22

**Published:** 2008-11-13

**Authors:** Konstantinos N Fountoulakis, Xenia Gonda, Zoltan Rihmer, Costas Fokas, Apostolos Iacovides

**Affiliations:** 1 Third Department of Psychiatry, Aristotle University of Thessaloniki, Thessaloniki, Greece; 2 Department of Clinical and Theoretical Mental Health, Kutvolgyi Clinical Center, Budapest, Hungary; 3 Department of Pharmacology and Pharmacotherapy, Semmelweis University, Faculty of Medicine, Budapest, Hungary; 4 First Department of Psychiatry, Aristotle University of Thessaloniki, Thessaloniki, Greece

## Abstract

**Background:**

Important methodological questions still exist concerning the Dexamethasone Suppression Test (DST), including the possibility of a better way of interpreting it. The aim of the present study was to explore the feasibility of an alternative way of interpreting DST results.

**Methods:**

A total of 50 patients with major depression aged 41.0 ± 11.4 years old participated in the study. Past and present suicide attempts were recorded. Psychometric assessment included the Hamilton Depression Rating Scale (HDRS), the Hamilton Anxiety Scale (HAS), the Newcastle Depression Diagnostic Scale (NDDS), the Diagnostic Melancholia Scale (DMS) and the General Assessment of Functioning (GAF) scale. The 1 mg DST protocol was used. Analysis methods included the chi square test and analysis of covariance (ANCOVA) with Fisher least significant difference (LSD) as post hoc tests.

**Results:**

In all, 34 patients (68%) were suppressors, 16 (32%) were non-suppressors and 14 patients had cortisol values above 5 μg/dl at baseline. Baseline cortisol level did not influence the classical DST interpretation. A total of 18 patients (36%) showed an increase of their cortisol levels after dexamethasone administration and 32 patients (64%) showed a decrease. Reducers had less melancholic features, similar levels of depression, better sleep and less suicidal thoughts in comparison to increasers. No relationship of DST to suicidality was found.

**Discussion:**

The present study explored the pattern of cortisol response to dexamethasone suppression and suggested an alternative way of coding and interpreting the DST on the basis of whether the cortisol levels remain stable or increase vs decrease after the administration of cortisol. The results put forward a complex way of understanding the relationship of the DST results with clinical symptoms.

## Introduction

Although the dexamethasone suppression test (DST) was first described as a biological marker for depression [1], it has also been associated with suicidal behaviour, melancholic and latter atypical features. Newer studies suggest that the hypothalamic-pituitary-adrenal (HPA) axis dysregulation shows different characteristics in suicidal and non-suicidal depressed patients [2] suggesting that DST status should also show a difference. More recently is has been also suggested that the DST response might differ even within the suicidal group, since in several studies DST non-suppression was associated with completed suicide but not with suicidal attempts [3,4]. This indicates that the relationship between the clinical manifestation and DST is more complex, and thus more attention should be paid to the investigation of this relationship and especially to the association of clinical symptoms with the different characteristics of DST.

The aim of the present study was to investigate the relationship between suicidal behaviour and the temporal characteristics of the DST. The temporal characteristics of the DST concerned whether non-suppression was defined on the basis of either of 16.00 or 23.00 day 2 cortisol levels, or both. The current report is complementary to a recently published paper by Yerevanian *et al*. [5]; however, these authors measured only the 16.00 cortisol level. The data in this brief report come from a larger research project on the neurobiology of depression, whose results have already been published [6]; however the DST characteristics reported here have not been published previously.

## Materials and methods

### Subjects

A total of 50 patients (15 males and 35 females) aged 21–60 years (mean 41.0, SD 11.4)) suffering from major depression according to the Diagnostic and Statistical Manual of Mental Disorders, 4th edition (DSM-IV) [7], took part in the study.

All participants provided written informed consent; 14 of them fulfilled criteria for atypical features, 16 for melancholic features and 32 for somatic syndrome (according to the International Classification of Diseases (ICD)-10). Nine patients did not fulfil criteria for any specific syndrome.

All participants were inpatients or outpatients of the Third Department of Psychiatry, Aristotle University of Thessaloniki, University Hospital AHEPA, Thessaloniki Greece and come from a study sample used in the PhD thesis of an author (KNF). The study on this specific population has already produced a significant number of publications [8-18].

All participants were free of any medication for at least 2 weeks prior to the first assessment and diagnosis. In no case was medication interrupted in order to include the patient in the study. In addition, all participants were physically healthy with normal clinical and laboratory findings, including electroencephalogram and thyroid function, and with no pathological findings from the opthalmological examination. There was a great effort to exclude all cases that might contribute to the production of confounding results due to special characteristics (for example, obesity, puerperium etc.). Additionally, a particular effort was made to exclude patients who exhibited alcohol or nicotine abuse.

No participant fulfilled the criteria for catatonic or psychotic features or for seasonal affective disorder. Additionally, no patient fulfilled the criteria for another DSM-IV axis-I disorder, except for generalised anxiety disorder and panic disorder, and none had a past history of manic or hypomanic episode. Axis II disorders were also registered.

All patients had history of no more than five distinct episodes including the present one (mean 1.16 ± 1.53).

It should be noted that all suicidal attempts were non-violent, and were performed by the swallowing of pills, drugs or poison. Rather, they had an impulsive character and no notes were prepared before attempts.

### Clinical diagnosis

The Schedules for Clinical Assessment in Neuropsychiatry version 2.0 (SCAN v 2.0))[19] and the International Personality Disorders Examination (IPDE) [20,21]were used to assist clinical diagnosis, which was reached by consensus of two examiners, according to DSM-IV criteria. The presence of a recent suicide attempt, the presence of an attempt ever in the past, the age of onset of depression, the number of episodes, the number of melancholic and atypical criteria met and the number of personality disorders diagnosed were recorded.

### Laboratory testing

Laboratory tests included blood and biochemical testing, test for pregnancy, T3, T4 and thyroid-stimulating hormone (TSH), Â12 and folic acid, and electroencephalogram.

### Psychometric assessment

Assessment included the Hamilton Depression Rating Scale (HDRS) and subscales [22-24], the Hamilton Anxiety Scale (HAS) and subscales [24,25], the 1965 Newcastle Depression Diagnostic Scale (1965 NDDS) [24] and 1971 Newcastle Depression Diagnostic Scale (1971 NDDS) [24], the Diagnostic Melancholia Scale (DMS) [24] and the General Assessment of Functioning scale (GAF)) [7]

On the basis of clinical and psychometric data, patients were divided into three groups. The first included those without death thoughts (no thoughts of death at all or wondering whether life has no meaning), the second included those with no specific thoughts about death (afraid that they will die or wish to die) and finally the third included truly suicidal patients (thinking about or planning suicide). We created a new categorical variable, named the Death Thoughts Rating, by attributing a score of 0 for members of the first group, 1 for the second and 2 for the third group; this is somewhat different from the rating of the HDRS item 3.

### Data concerning personal and family history and stressful life events

The family history method [26-28] was used for gathering family and personal data. The Holmes questionnaire [29]was used to search for stressful life events during the 6 months prior to the onset of the symptomatology. The patients were carefully questioned concerning the presence of at least one first-degree predecessor with any type of dementia.

### Dexamethasone Suppression Test

The DST [1,30-35] mainly reflects the HPA axis and norepinephrine activity. The 1 mg DST protocol demands the administration of 1 mg dexamethasone taken orally at 23.00 on the first day, and determination of cortisol serum levels simultaneously and the next day at 16.00 and 23.00. Cortisol levels expressed in μg/dl were measured by luminance immunoassay (intra-assay reliability: 4.9%; interassay: 7.5%). Non-suppression cut-off level was 5 μg/dl.

### Statistical analysis

The following grouping methods were used:

• The two classical groups, that is suppression vs non-suppression on the basis of the 5 μg/dl cut-off level.

• Two groups on the basis of whether cortisol values increased or decreased after dexamethasone administration (increasers vs decreasers)

• Groups defined by the interaction of the above two grouping methods.

• Two groups defined by the presence or not of a recent suicide attempt (within the last month).

• Two groups defined by the presence or not of a suicide attempt ever in the past

• Three groups defined by the Death Thoughts Rating variable (A: no thoughts of death, B: non-specific thoughts of death and C: suicidal ideation).

These patient groups were compared using:

• Crosstabulation frequencies between groups using the chi square test.

• Three analysis of covariance (ANCOVA) tests were performed, with the two ways to define DST results as grouping variable (in a yes/no format) and a third with their combination, and the following as dependent variables: age at onset, number of episodes, number of melancholic and atypical criteria met, GAF, HDRS (and its subscales: depressed index, anxiety index, non-specific index and sleep index) [24], HAS (and its subscales: somatic and psychic anxiety) [24], the 1965 NDDS and 1971 NDDS the DMS (with its subscales: endogenous and reactive axis [24], number of total events, number of personality disorders diagnosed, and Death Thoughts Rating (as described above). In total, 20 variables were used. Age was used as covariate. Because of the number of ANCOVAs performed, the p < 0.016 level was used to define significance. Age was used as covariate.

The Fisher least significant difference (LSD) test was used for the post hoc comparisons. For these comparisons the p < 0.05 level was used to define significance.

## Results

In all, 34 patients (68%) were suppressors and 16 (32%) were non-suppressors with the use of the 5 μg/dl cut-off point. It is to be noted that 14 (28%) patients had cortisol values above 5 μg/dl at baseline. The results of the DST test with the patients categorised according to their baseline cortisol values (above and below 5 μg/dl) are shown in table 1. It is evident that the baseline cortisol level does not seem to influence the classical DST interpretation (chi square test, degrees of freedom = 1, p = 0.723).

**Table 1:** Dexamethasone Suppression Test (DST) results with patients categorised according to their baseline cortisol values

	**Suppressors**	**Non-suppressors**
Baseline cortisol < 5 μg/dl (n = 36):		

Day 2, 16:00	32 (88.88%)	4 (11.12%)

Day 2, 23:00	27 (75%)	9 (25%)

Either at 16:00 or 23:00	25 (69.44%)	11 (30.56%)

Baseline cortisol > 5 μg/dl (n = 14):		

Day 2, 16:00	9 (64.28%)	5 (35.72%)

Day 2, 23:00	12 (85.71%)	2 (14.29%)

Either at 16:00 or 23:00	9 (64.28%)	5 (35.72%)

Overall (n = 50)	34 (68%)	16 (32%)

Some patients showed an increase of their cortisol levels after dexamethasone administration while others experienced a reduction. The respective frequencies are shown in table 2. There was no effect from baseline cortisol levels (chi square test, degrees of freedom = 1, p = 0.633). In all, 18 patients (36%) showed an increase of their cortisol levels. This group overlaps significantly with classical non-suppression (table 3). However, for 12 patients (24%) the two different classifications were not identical.

**Table 2:** Changes in cortisol levels after dexamethasone administration

	**Day 2, 16:00**	**Day 2, 23:00**	**Either or both**
Baseline cortisol < 5 μg/dl (n = 36):			

Increased or unchanged	12 (33.34%)	13 (30.56%)	14 (38.88%)

Increased	6 (16.67%)	10 (27.78%)	

Unchanged	6 (16.67%)	1 (2.78%)	

Decreased	24 (66.66%)	25 (69.44%)	

Baseline cortisol > 5 μg/dl (n = 14):			

Increased or unchanged	4 (28.57%)	1 (7.14%)	4 (28.57%)

Increased	4 (28.57%)	1 (7.14%)	

Unchanged	0 (0%)	0 (0%)	

Decreased	10 (71.43%)	13 (92.86%)	

Overall (n = 50):			

Increased or unchanged	16 (32%)	12 (24%)	18 (36%)

Increased	10 (20%)	11 (22%)	

Unchanged	6 (12%)	1 (2%)	

Decreased	34 (68%)	38 (76%)	

**Table 3:** Relationship between increasers vs reducers and non-suppressors vs suppressors

	**Non-suppressors**	**Suppressors**	**Total**
Increasers	15	3	18

Decreasers	1	31	32

Total	16	34	50

On testing the two different DST classifications with ANCOVA, the results were as follows: for the classical DST non-suppression (either at 16.00 or at 23.00) the ANCOVA showed a significant effect (Wilks L = 0.199; F = 5.632, effect = 20; error = 28; p = 0.00002). The post hoc results are shown in table 4. For the increaser/reducer DSTs (three groups: reducers, increased levels only once, increased both levels), the ANCOVA showed a significant effect (Wilks L = 0.113; F = 2.651; effect = 40; error = 54; p = 0.0004). The post hoc results are shown in table 5.

**Table 4:** Analysis of covariance (ANCOVA) post hoc test for the classical Dexamethasone Suppression Test (DST) definition

	**DST suppressors**		**DST non-suppressors**		**p Value**
	
	**Mean**	**SD**	**Mean**	**SD**	
GAF	48.74	13.97	58.13	13.65	0.021

DMS Endogenous axis	3.88	2.92	6.13	3.03	0.011

HDRS Depressive Index	10.97	2.76	8.63	2.31	0.005

Death Thoughts Rating	1.00	0.70	0.56	0.73	0.044

DMS, Diagnostic Melancholia Scale; GAF, General Assessment of Functioning; HDRS, Hamilton Depression Rating Scale.

**Table 5:** Analysis of covariance (ANCOVA) post hoc test for the reducers/increasers Dexamethasone Suppression Test (DST) definition

							**Fisher least significant difference (LSD) test, p value**		
	**Reducers (R)**		**Single level Inreasers (SI)**		**Both levels Increasers (BI)**		**R vs SI**	**R vs BI**	**SI vs BI**
				
	**Mean**	**SD**	**Mean**	**SD**	**Mean**	**SD**			

No of atypical features	0.94	0.98	1.53	0.74	1.00	1.00	0.037	NS	NS

No of melancholic features	2.25	1.52	2.20	1.61	4.00	1.73	NS	0.046	NS

HDRS	26.38	5.97	23.00	3.93	29.00	3.61	0.047	NS	NS

HDRS Depressive Index	10.84	2.93	8.87	2.33	10.33	2.08	0.026	NS	NS

HDRS Sleep Index	3.41	2.11	3.47	1.46	6.00	0.00	NS	0.025	0.036

Death Thoughts Rating	1.00	0.72	0.40	0.51	1.67	0.58	0.005	0.003	NS

Baseline cortisol	5.16	5.70	4.18	3.23	3.80	2.75			

Cortisol day 2, 16:00	1.94	4.29	3.77	3.89	7.17	1.46			

Cortisol day 2, 23:00	1.58	2.71	5.16	3.65	7.00	1.66			

HDRS, Hamilton Depression Rating Scale; NS, not significant.

For the combination of the two DST interpreting methods into a single grouping variable with three levels (suppressors and reducers vs non-suppressors and increasers vs other combinations) the ANCOVA showed again a significant effect (Wilks L = 0.129; F = 2.403; effect = 40; error = 54; p = 0.001). The post hoc results are shown in table 6.

**Table 6:** Analysis of covariance (ANCOVA) post hoc test for the combined Dexamethasone Suppression Test (DST) definition

							**Fisher least significant difference (LSD) test, p value**		
	**Suppressors and reducers (SR)**		**Other (O)**		**Non-suppressors and increasers (NSI)**		**SR vs O**	**O vs NSI**	**SR vs NSI**
				
	**Mean**	**SD**	**Mean**	**SD**	**Mean**	**SD**			

N1971	-5.02	19.30	-0.54	14.69	-19.55	18.44	NS	0.013	0.026

End DMS	3.82	3.02	4.17	2.37	7.30	2.87	NS	0.007	< 0.001

HDRS depressive index	11.00	2.84	9.00	2.76	9.50	2.32	0.041	NS	NS

DMS, Diagnostic Melancholia Scale; HDRS, Hamilton Depression Rating Scale; NS, not significant.

The comparison of the relationship the two DST definitions have with suicidality and thoughts of death is shown in tables 7 and 8.

**Table 7:** Dexamethasone Suppression Test (DST) suppression vs non-suppression and increasers vs reducers rates in the different suicidal behaviour groups, Yates corrected chi square tests

	**Suppressors (n = 34)**		**Non-suppressors (n = 16)**		**Chi square**	**Increasers**		**Reducers**		**Chi square**
	**n**	**%**	**n**	**%**	**p Value**	**n**	**%**	**n**	**%**	**p Value**

Recent suicide attempt	5	14.70	0	0.00	0.266	2	11.11	3	9.37	0.844

Ever suicide attempt	11	32.35	2	12.50	0.135	4	22.22	8	25.00	0.825

Death thoughts rating:					0.074					0.305

No death thoughts	8	23.53	9	56.25		6	33.33	11	34.37	

Non-specific thoughts of death	18	52.94	5	31.25		6	33.33	16	50.00	

Suicidal ideation	8	23.53	2	12.50		6	33.33	5	15.62	

**Table 8:** Combined Dexamethasone Suppression Test (DST) definitions in the different suicidal behaviour groups, Yates corrected chi square tests

	**Suppressors and reducers (SR) (n = 28)**		**Other (O) (n = 12)**		**Non-suppressors and increasers (NSI) (n = 10)**		**Chi square, p-value**		
	**n**	**%**	**n**	**%**	**n**	**%**	**SR vs O**	**O vs NSI**	**SR vs NSI**

Recent suicide attempt	3	10.71	2	16.66	0	0.00	0.602	0.542	0.692

Ever suicide attempt	9	32.14	2	16.66	2	20.00	0.315	0.723	0.385

Death thoughts rating:							0.573	0.851	0.312

No death thoughts	7	25.00	5	41.67	5	50.00			

Non-specific thoughts of death	15	53.57	5	41.67	3	30.00			

Suicidal ideation	6	21.43	2	16.67	2	20.00			

## Discussion

The present study explored the pattern of cortisol response to dexamethasone suppression and suggested an alternative way of coding and interpreting the DST on the basis of whether the cortisol levels remain stable or increase vs decrease after the administration of cortisol. In the current study sample, 34 patients (68%) were suppressors and 16 (32%) were non-suppressors with the use of the classic 5 μg/dl cut-off point. The use of both the 16:00 and 23:00 day 2 cortisol values seems to be important otherwise significant false results appear. With the use of the new suggested increasers/reducers dexamethasone test (IRDT), 18 patients (36%) had unchanged or showed an increase of their cortisol levels vs 32 patients (64%) who showed a decrease. The two classification methods overlap significantly but they are not identical and for 24% of patients are classified differently. Although several patients had high (above 5 μg/dl) baseline cortisol values, it seems that this does not influence the post dexamethasone values.

The classical DST interpretation leads to the conclusion that DST suppressors are patients that are less endogenous, with lower functioning, and with higher suicidal thoughts in comparison to non-suppressors. These results are by themselves peculiar and do not fit with the classical definition and concept of depression. By comparison, reducers had less melancholic features, similar levels of depression, better sleep and less suicidal thoughts in comparison to increasers. Suppressors and reducers were less endogenous and with lower depression in comparison to non-suppressors and increasers. Since anxiety was not assessed in the current study but several HDRS items include a strong anxiety component, and the HDRS anxiety index was calculated, it is unlikely that the presence of anxiety is hidden behind the peculiarity of results. However, it is possible that the combined use of the two methods to interpret the DST could isolate a subgroup of depressed patients at high risk to show suicidal behaviour.

Our current study reported that 10% of depressed patients from the study sample had recently attempted suicide and 26% had attempted at least once in the past. Additionally, 46% were experiencing some kind of thoughts of death and 20% were experiencing suicidal ideation at interview time. Concerning suicidality, no method reported in the current study, showed a strong potential in correlating with history of suicidal acts or present suicidal ideation.

DST non-suppression has originally been associated with the endogenous, melancholic and psychotic type of depression [36] and has been described as a highly state dependent marker characteristic of the symptomatic phase of the disorder [37,38]. Our results are in line with these observations and indicate that non-suppressors in our sample score significantly higher on the endogenous axis of the DMS. DST non-suppression in our study, however, is also related to a higher general functioning score (GAF), and lower scores on the depressed index of the HDRS and in case of the death thoughts rating. This is true when we treat all patients showing any DST non-suppression as one group; however, distinctions within the DST non-suppressors emerge if we form separate groups according to the temporal characteristics of DST suppression. Therefore our study indicates that the temporal characteristics of DST should be taken into account when outlining the relationship between DST and suicidal behaviour.

Most previous studies conducted on the association of DST non-suppression and suicidal behaviour concluded that DST non-suppression is related to suicidal ideation and to future suicide rather than committed suicide [3,39]. However, most of the studies coming to this conclusion use only the 23:00 on day 2 cortisol levels in order to define DST non-suppression. The results of the current study, however, suggest that the picture is more complex. Recent data suggest that patients with a history of suicidal behaviour suffer a greater burden of depressive illness [40] and this could be reflected in the DST results.

DST suppression has been associated previously with suicide [4,39,41,42], and it has also been suggested that abnormal DST has a differential relationship with different types of suicidal behaviour although this relationship is weak [3,43].

Investigating the different non-suppressor groups with distinct temporal characteristics and in relation to absolute cortisol levels could give deeper insight into the associations of DST and characteristics of depression and suicidal behaviour. However, further research on larger numbers is necessary in this area, although it does appear promising.

Compared to the beginnings of research on DST when it was conceived as being related to endogenous and melancholic depression [1,36], DST results according to more contemporary research seem to suggest it is a severity marker rather than directly related to symptomatology. Nearly 4–10% of normal persons are reported to be DST non-suppressors [44-46]. The reason for this is unknown, however it has been suggested that it is due to an underlying mood disorder or family history of affective disorder. Another explanation suggests that DST reflects in fact the degree of psychological pressure or discomfort of the subject and not a specific vulnerability or characteristic of depression [47,48]. It seems that non-suppression is gradually increasing along a continuum, which has mourning outpatients at one pole (13% non-suppression) and severe psychotic melancholic inpatients with psychotic features and suicidal ideation at the opposite one (64% non-suppression) [49]. In this frame, the percentage of non-suppression reported in the current study (32%, with all types of non-suppression combined) is not in contrast with the international literature, since most patients were outpatients. However, if DST non-suppression can be characterised as being related to different severities of mood disorders along a continuum, then it is also worth considering whether DST non-suppression should be characterised in a more sophisticated way with attention paid to its temporal characteristics, rather than operating with only two groups (namely DST suppression and non-suppression). In some studies different plasma cortisol threshold levels are already being used to investigate more deeply the relationship between DST non-suppression with depression or suicide [50]. These more elaborate classifications of DST non-suppression would allow us to use DST to map different subtypes of depression and suicidal behaviour in a more sensitive and complex way.

## Conclusion

The results of our study, especially in the light of the previous literature on DST, indicate that the relationship between DST and clinical symptomatology is very complex, and also that methodological problems and contradictions often halt the possibility of drawing all data and conclusions from studies and of comparing the results. Specific characteristics of the response to dexamethasone administration should be taken into account in a more complex way when analysing the relationship between DST and clinical symptoms in order to avoid missing a possible existing relationship.

## Competing interests

The authors declare that they have no competing interests.

## Authors' contributions

KNF designed the protocol, gathered and analysed the data and contributed to interpretation and the draft and the final paper. XG contributed in the analysis of the data and to interpretation and the draft and the final paper. ZR contributed in the analysis of the data and to interpretation and the draft and the final paper. CF and AI contributed in the design of the protocol, the gathering of the data and the final paper.
